# Advanced Predictive Analytics for Fetal Heart Rate Variability Using Digital Twin Integration

**DOI:** 10.3390/s25051469

**Published:** 2025-02-27

**Authors:** Tunn Cho Lwin, Thi Thi Zin, Pyke Tin, Emi Kino, Tsuyomu Ikenoue

**Affiliations:** 1 Interdisciplinary Graduate School of Agriculture and Engineering, University of Miyazaki, Miyazaki 889-2192, Japan; z322t02@student.miyazaki-u.ac.jp; 2 Graduate School of Engineering, University of Miyazaki, Miyazaki 889-2192, Japan; pyketin11@gmail.com; 3 Faculty of Medicine, University of Miyazaki, Miyazaki 889-1692, Japan; emi_kino@med.miyazaki-u.ac.jp (E.K.); tsuyomu_ikenoue@med.miyazaki-u.ac.jp (T.I.)

**Keywords:** approximate entropy, fetal heart rate, hidden Markov model, predictive analytics, umbilical cord blood gas

## Abstract

Fetal heart rate variability (FHRV) is a critical indicator of fetal well-being and autonomic nervous system development during labor. Traditional monitoring methods often provide limited insights, potentially leading to delayed interventions and suboptimal outcomes. This study proposes an advanced predictive analytics approach by integrating approximate entropy analysis with a hidden Markov model (HMM) within a digital twin framework to enhance real-time fetal monitoring. We utilized a dataset of 469 fetal electrocardiogram (ECG) recordings, each exceeding one hour in duration, to ensure sufficient temporal information for reliable modeling. The FHRV data were preprocessed and partitioned into parasympathetic and sympathetic components based on downward and non-downward beat detection. Approximate entropy was calculated to quantify the complexity of FHRV patterns, revealing significant correlations with umbilical cord blood gas parameters, particularly pH levels. The HMM was developed with four hidden states representing discrete pH levels and eight observed states derived from FHRV data. By employing the Baum–Welch and Viterbi algorithms for training and decoding, respectively, the model effectively captured temporal dependencies and provided early predictions of the fetal acid–base status. Experimental results demonstrated that the model achieved 85% training and 79% testing accuracy on the balanced dataset distribution, improving from 78% and 71% on the imbalanced dataset. The integration of this predictive model into a digital twin framework offers significant benefits for timely clinical interventions, potentially improving prenatal outcomes.

## 1. Introduction

Fetal heart rate variability (FHRV) is a critical parameter in obstetrics, serving as a key indicator of fetal well-being and autonomic nervous system (ANS) development [1]. However, traditional monitoring methods provide limited insights, often resulting in delayed interventions and suboptimal outcomes [2]. Advanced predictive analytics applied to FHRV allow for the extraction of subtle patterns and trends that conventional analysis may not detect. Machine learning models, such as neural networks and the hidden Markov model (HMM), can process vast amounts of fetal heart rate data, identifying deviations that may indicate fetal distress or developmental concerns [3]. This predictive capability is crucial for early interventions, potentially reducing the incidence of complications such as hypoxia or preterm birth.

Digital twin technology further enhances predictive analytics by creating a virtual representation of the fetal physiological environment. This model simulates clinical scenarios, allowing clinicians to anticipate fetal responses to interventions and optimize care strategies accordingly [4,5]. By integrating diverse physiological data, digital twins facilitate real-time FHRV analysis without posing risks to the mother or fetus.

FHRV, measured through electrocardiogram (ECG) devices [6,7] reflects the activity of the autonomic nervous system (ANS) [8,9], which includes sympathetic and parasympathetic branches [10,11,12,13]. The sympathetic nervous system increases the heart rate in response to stress, while the parasympathetic nervous system decreases the heart rate to promote relaxation. By analyzing the balance between these systems through FHRV, healthcare providers can gain important insights into the fetus’s response to the stresses of labor, allowing for timely and appropriate interventions [14,15].

Despite the importance of FHRV monitoring, it does not directly measure the fetal acid–base status or respiratory function. Umbilical cord blood gas parameters, such as pH, pressure of carbon dioxide (PCO_2_), partial pressure of oxygen (PO_2_), bicarbonate (HCO_3_), and base excess (BE), are critical indicators of these functions, but are only available postnatally [16,17,18]. Therefore, there is a need for predictive models that can estimate these parameters in real time using non-invasive FHRV data. Analyzing these blood gas parameters helps assess the fetal respiratory status and detect any potential complications [19]. However, this information is only available postnatally, limiting its utility for intrapartum interventions.

To address this gap, we propose an advanced predictive analytics approach utilizing HMMs. HMMs are statistical models well-suited for handling time-series data and capturing hidden states representing underlying physiological processes [20]. These models effectively analyze sequential data where the system is assumed to follow a Markov process with unobserved states. By integrating HMM with FHRV data, this predictive framework can provide clinicians with immediate insights into fetal acid–base balance and respiratory function, eliminating the delay associated with post-delivery blood gas analysis. The early identification of potential complications, such as hypoxia or acidosis, would enable timely interventions and potentially improve neonatal outcomes [21].

The remainder of this paper is organized as follows: Section 2 reviews related work. Section 3 details the proposed methodology, while Section 4 presents the experimental results, evaluation of the predictive model, and discusses the findings. Finally, Section 5 offers conclusions and suggestions for future work.

## 2. Related Work

Research on FHRV has advanced significantly over recent decades, highlighting its crucial role as an indicator of fetal well-being and autonomic nervous system development [22]. Traditional analysis methods have predominantly utilized time-domain metrics like the standard deviation of normal-to-normal (SDNN) intervals and frequency-domain metrics such as the low-frequency to high-frequency (LF/HF) power ratio [23]. While valuable, these methods often fail to capture the complex, nonlinear dynamics inherent in fetal heart rate patterns.

To address these limitations, advanced analytical techniques, including nonlinear dynamics and entropy-based measures, have been explored to extract more informative features from FHRV data [24,25]. For instance, approximate entropy has been used to quantify the unpredictability of FHRV, providing deeper insights into autonomic regulation [26].

The application of predictive analytics to FHRV has shown considerable promise in enhancing the early detection of fetal distress. Machine learning models such as support vector machines, neural networks, and decision trees have been employed to classify fetal states and predict adverse outcomes [27,28]. The authors of [29] demonstrated that neural networks could improve the prediction of fetal hypoxia by analyzing complex FHRV patterns. However, these approaches often require large, high-quality datasets and may not fully account for temporal dependencies and hidden physiological states influencing FHRV signals.

Hidden Markov models (HMMs) have been utilized in biomedical signal processing due to their ability to model time-series data and capture hidden states representing underlying physiological processes [20]. In the context of FHRV, the authors of [30] employed autoregressive HMMs to detect fetal distress by identifying changes in heart rate variability associated with various physiological conditions. While effective in handling sequential data, the integration of HMMs with real-time fetal monitoring remains limited by computational complexity and the need for real-time data processing capabilities.

Digital twin technology has emerged as a transformative approach in healthcare, offering dynamic, virtual replicas of physical entities to simulate and predict system behaviors [31]. In obstetrics, digital twins of the fetal environment enable clinicians to visualize and analyze complex physiological interactions in real time [32]. Initial studies have demonstrated the potential of fetal digital twins in enhancing prenatal care by providing a more comprehensive understanding of fetal physiology [33]. However, integrating digital twins with advanced predictive analytics, such as HMMs, for real-time fetal monitoring is still in its early stages and presents an opportunity for innovation.

Several studies have investigated the correlation between FHRV and umbilical cord blood gas parameters to enhance the assessment of fetal well-being. The authors of [34] found that certain FHRV metrics correlated with blood gas parameters like pH and base excess (BE), critical indicators of the fetal acid–base status and respiratory function. These findings suggest that FHRV analysis could potentially predict adverse metabolic conditions before delivery. However, most of these studies have been retrospective and lack models capable of providing real-time predictions during labor. The challenge lies in accurately mapping the complex relationship between FHRV patterns and metabolic states, influenced by factors such as fetal activity, gestational age, and maternal health [35].

Despite advancements in FHRV analysis, predictive modeling, and digital twin technology, there remains a gap in integrating these technologies to provide real-time, non-invasive assessments of the fetal acid–base status during labor. Existing research has primarily focused on each component individually, without fully leveraging their combined potential. The innovative integration proposed in this study aims to address this gap by developing a predictive model using HMMs within a digital twin framework. This approach has the potential to enhance clinical decision-making by providing immediate insights into the fetal condition, allowing for timely interventions and potentially improving prenatal outcomes.

## 3. Materials and Methods

This section outlines the comprehensive methodology adopted to enhance fetal monitoring by integrating digital twin technology with entropy-based fetal heart rate variability (FHRV) analysis during delivery. The proposed approach leverages digital twin models to create a virtual representation of the fetal cardiovascular system, enabling the advanced analysis and prediction of fetal heart rate patterns. The methodology includes data acquisition, the development of the digital twin model, data preprocessing, dataset partitioning, approximate entropy calculation, correlation analysis, and the application of an HMM for prediction.

### 3.1. Data Acquisition

**Figure 1:**
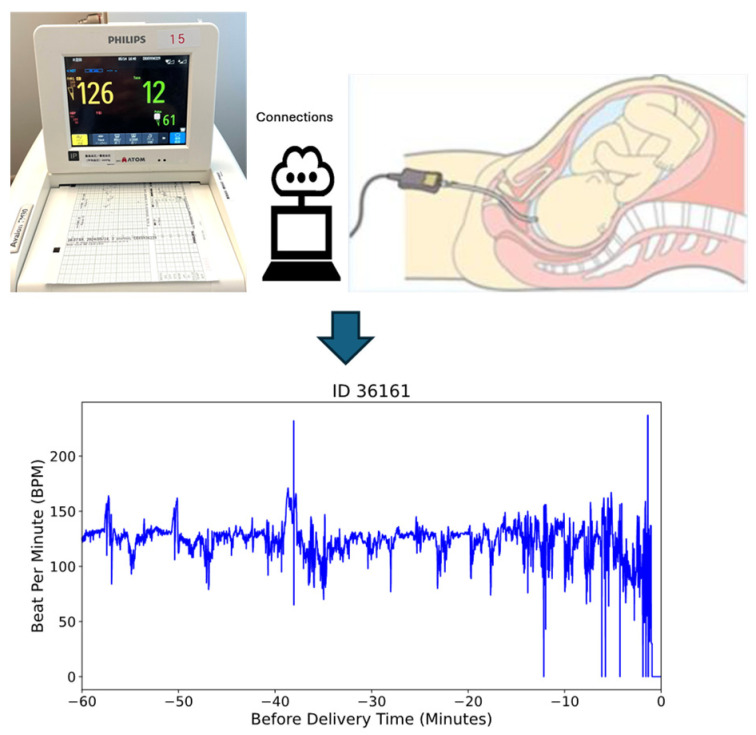
The internal monitored beat per minute (bpm) data during delivery time.

In this study, we utilized a dataset comprising 585 fetal heart rate (FHR) recordings obtained through internal electrocardiogram (ECG) monitoring during labor at full-term gestation (≥37 weeks). To ensure data quality and consistency, we selected recordings with durations of longer than one hour, resulting in a final dataset of 469 recordings for analysis. This criterion was applied to capture sufficient temporal information for reliable modeling and analysis. Figure 1 illustrates the internal monitoring setup, where a scalp electrode is attached directly to the fetal scalp to capture real-time FHR data [36].

These recordings were collected at Miyazaki Medical Association Hospital, Miyazaki Prefecture, Japan from 2017 to 2022. Ethical approval was obtained from the Miyazaki Medical Association Hospital’s Institutional Review Board, and informed consent was secured from all participants.

### 3.2. Development of Digital Twin Model

We developed a digital twin model of the fetal cardiovascular system using MATLAB R2024a, integrating physiological parameters extracted from fetal heart rate (FHR) data, including FHRV and ANS responses. This virtual model simulates fetal responses under various conditions, enabling the advanced analysis and prediction of heart rate patterns. The digital twin bridges the physical and virtual worlds, providing a real-time, non-invasive, and adaptive solution for maternal–fetal care [31].

The model employs a hidden Markov model (HMM) to analyze sequential changes in FHR patterns, facilitating the early detection of the fetal acid–base status. The HMM structure consists of four hidden states, representing different fetal acid–base conditions based on clinically defined pH thresholds. The transition matrix is trained on observed fetal state transitions, while emission probabilities are derived from FHRV feature distributions. The model is trained using the Baum–Welch algorithm and decoded via the Viterbi algorithm to classify fetal states dynamically. Hidden state categorization was refined through statistical clustering and medical consultation, ensuring clinically meaningful classifications.

**Figure 2:**
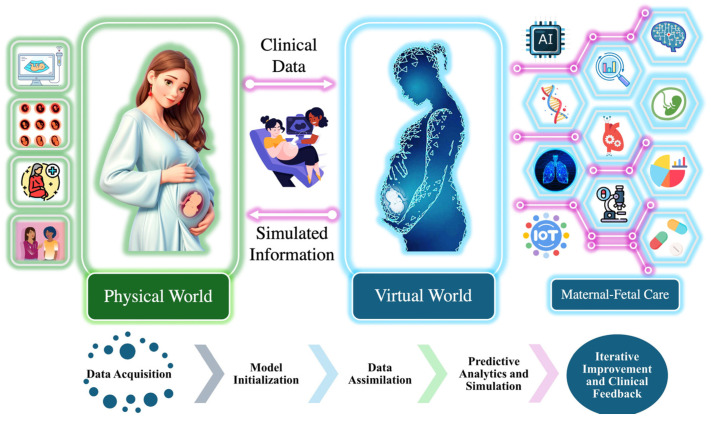
Integration of physical and virtual world for maternal–fetal care.

As illustrated in Figure 2, real-time data from the physical world are collected using maternal–fetal monitoring devices such as ultrasound, wearable sensors, and fetal scalp electrodes, which are shown in in Figure 1. These inputs are transmitted into the virtual world via IoT-based systems, forming a real-time digital replica of the maternal–fetal environment [37,38,39,40].

Within the digital twin environment, the system follows a structured workflow comprising model initialization, data assimilation, predictive analytics and simulation, and iterative improvement and clinical feedback. Model initialization involves establishing the baseline physiological parameters, creating a virtual representation of fetal cardiovascular dynamics. Data assimilation is conducted to ensure that real-time FHRV data are continuously integrated into the model, enabling dynamic updates that reflect real-world conditions. Predictive analytics and simulation involve utilizing the HMM to analyze sequential changes in FHR patterns, detect state transitions in the fetal acid–base status, and predict potential adverse outcomes. Finally, the iterative improvement and clinical feedback loop refines the model over time by incorporating new clinical insights and data, ensuring the continuous enhancement of predictive performance and decision support.

The digital twin continuously updates based on real-world inputs, enabling clinicians to visualize and analyze fetal physiological states in real time. Its predictive capability is validated by comparing simulated FHRV responses with clinical fetal monitoring data, ensuring that the model accurately reflects physiological trends. By integrating computational modeling with real-time fetal monitoring, the digital twin facilitates the early detection of fetal hypoxia, supports personalized intervention strategies, and provides a dynamic simulation environment to assess fetal responses under various clinical conditions.

**Figure 3:**
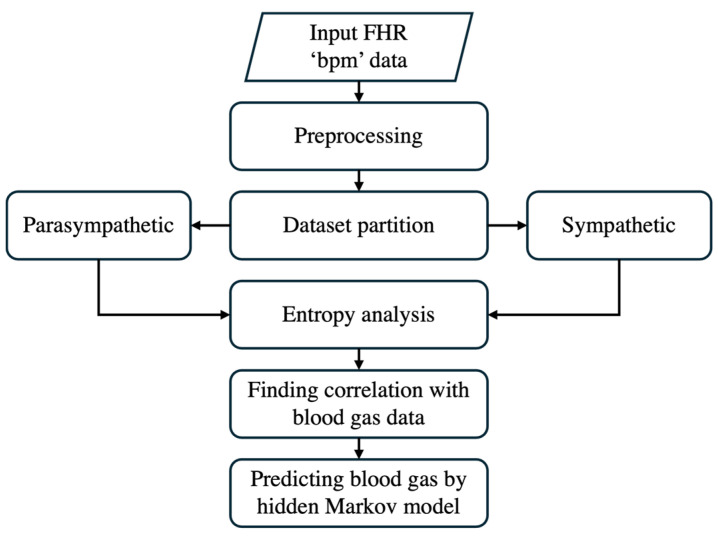
The step-by-step approach of predictive analytics.

The framework outlined in Figure 3 illustrates the predictive analytics workflow within the digital twin environment. This figure focuses specifically on how real-time maternal and fetal data are processed to develop and apply predictive models for fetal monitoring. The flowchart highlights key stages, including data acquisition, preprocessing, model assimilation, predictive analytics, and simulation, demonstrating how raw physiological signals are transformed into actionable clinical insights. By structuring the predictive component within the digital twin, this approach enhances prenatal care decision-making by ensuring continuous adaptation to fetal conditions and enabling timely interventions to improve prenatal outcomes.

### 3.3. Preprocessing

When preprocessing FHRV data in beats per minute (bpm), we employed a method for cleaning the data by removing outliers. This process involves calculating the average (μ) and standard deviation (σ) of the bpm data. We retained data points within the range defined by Equation (1):

(1) $$\mu -3\sigma \leq {\mathrm{FHR}}_{\mathrm{bpm}}\leq \mu +3\sigma$$

where FHR_bpm_ represents the fetal heart rate in bpm. This range captures approximately 99.7% of data in a normal distribution, ensuring that extreme values, which may distort the analysis, are excluded. Importantly, removed data points are omitted and not replaced with any artificial values.

**Figure 4:**
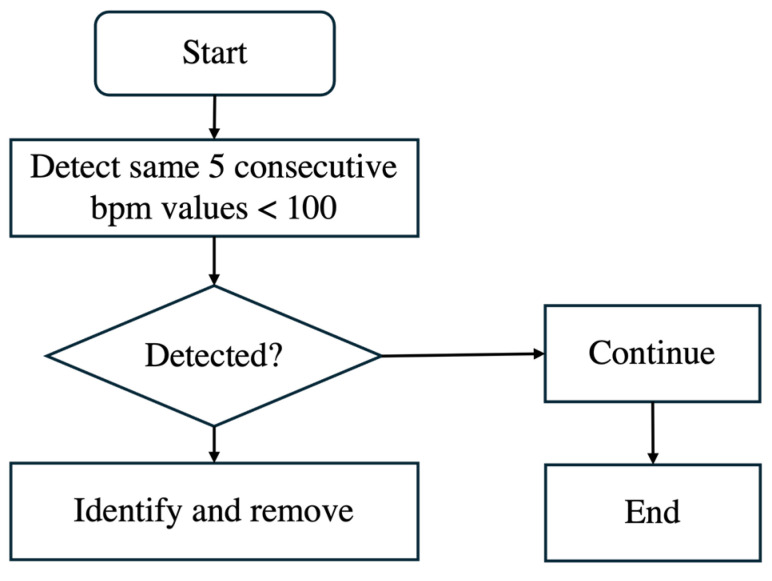
Flowchart of removing noise bpm.

Additionally, noise is often present after delivery because, once the baby is born, the scalp electrode is removed. However, the recording machine does not stop automatically and continues recording, leading to unusable data. This noise is characterized by bpm values significantly slowing down, falling below 100 bpm, and occurring consecutively. Such values, if left unprocessed, can distort the analysis. As shown in the flowchart of Figure 4, we identified five consecutive bpm values lower than 100 bpm as noise and removed them from the dataset.

**Figure 5:**
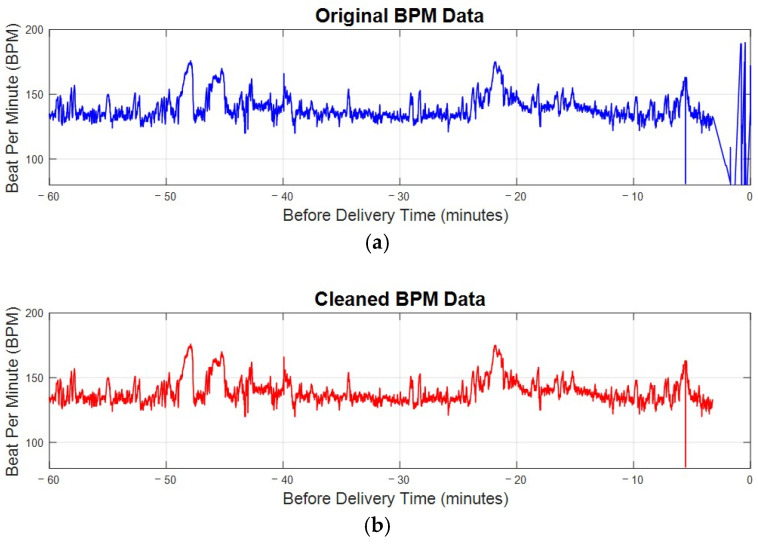
The bpm plot of FHR before and after preprocessing. (**a**) The original bpm plot of FHR directly received from electrode. (**b**) The cleaned bpm data after preprocessing.

Figure 5 compares a sample patient’s original bpm data in Figure 5a with the cleaned bpm data in Figure 5b. In the original data, irregularities and significant noise are noticeable, particularly toward the end of the recording period, where bpm values drop sharply due to post-delivery noise. The cleaning process, outlined in the algorithm in Figure 4, effectively removes these outliers and noise. The result is a smoother and more consistent data trend. By focusing only on valid and representative bpm data, this preprocessing step ensures that subsequent analyses are more accurate and reliable, eliminating distortions caused by unusable data points. This step is essential for maintaining the integrity of FHRV analysis, particularly when working with sensitive bpm data.

### 3.4. Dataset Partition

The dataset was divided into two components representing parasympathetic and sympathetic activities, based on the direction of heart rate changes. The classification of parasympathetic activity as “downward” and sympathetic activity as “non-downward” is critical for understanding fetal autonomic responses during delivery. Downward beats, where the heart rate decreases by a specific threshold, are associated with parasympathetic activation, reflecting the body’s mechanisms for slowing the heart rate and maintaining balance. Non-downward beats correspond to sympathetic activity, indicating an increased heart rate in response to stress.

Equation (2) illustrates the mathematical approach used for detecting downward beats in FHR data:

(2) $$\mathrm{Classification}:\left\{\begin{array}{l} {\mathrm{Downward},\;\mathrm{FHR}}_{i+1}\leq {\mathrm{FHR}}_{i}-5\;\mathrm{bpm}\\ \mathrm{Non}\text{-}\mathrm{downward},\;\mathrm{otherwise} \end{array}\right.$$

where ${\mathrm{FHR}}_{i}$ represents fetal heart rate in bpm at the $i^{th}$ time point of the corresponding time. The threshold of 5 bpm was selected based on previous research that applied this value to identify significant FHR changes, including artifact detection, where values exceeding this threshold were considered physiologically relevant and required correction [41]. Additionally, consultation with medical professionals in this study confirmed that this threshold aligns with clinical assessments of parasympathetic activation and its impact on fetal heart rate regulation. This combined evidence supports the use of a 5 bpm threshold as a meaningful indicator of autonomic nervous system responses in FHR analysis.

**Figure 6:**
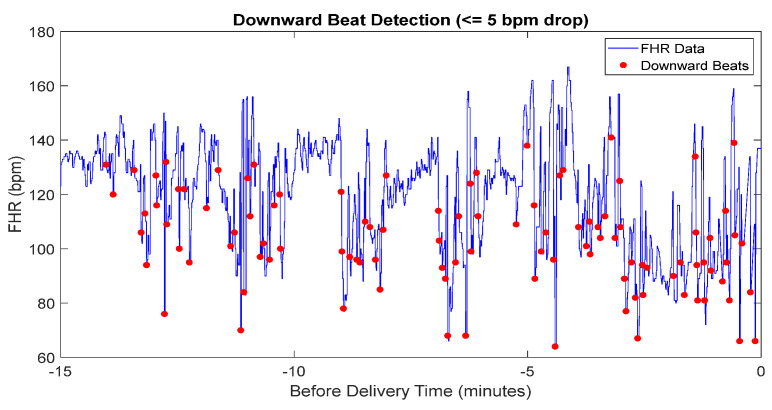
The detected downward beat of FHR data.

The results of downward beat detection, illustrated in Figure 6, show the differentiation between parasympathetic and sympathetic activity during labor. Downward beats are marked where the heart rate drops by 5 bpm or more compared to the previous beat. By distinguishing these events, this analysis provides deeper insight into the FHR and enhances the assessment of fetal well-being.

### 3.5. Approximate Entropy

Approximate entropy (ApEn) is a statistical measure that quantifies the complexity and predictability of fluctuations in FHRV by assessing the logarithmic likelihood of patterns in time-series data [42]. It differentiates between regular and chaotic patterns, providing valuable insights into autonomic nervous system dynamics, which are critical for predicting adverse outcomes [43].

The ApEn calculation involves the following steps:

(1) Construct vectors:

(3) $$x(i)=[u(t_{i}),u(t_{i+1}),\ldots ,u(t_{i+m-1})]$$

where $u(t_{i})$ is bpm at time $t_{i}$ and *m* is the embedded dimension.

(2) Calculate the Chebyshev distance between vectors:

(4) $$d[x\left(i\right),x\left(j\right)]=\max\left(\left|u(t_{i+k-1})-u(t_{j+k-1})\right|,1\leq k\leq m\right)$$

(3) Calculate $C_{i}^{m}(r)$ for each i, 1≤i≤N−m+1:

(5) $$C_{i}^{m}(r)=\frac{\mathrm{number}\;\mathrm{of}\;j\;\mathrm{such}\;\mathrm{that}\;d\left[x(i),x(j)\right]\leq r}{N-m+1}$$

(4) Compute $\Phi^{m}(r)$:

(6) $$\Phi^{m}(r)=\frac{1}{N-m+1}\sum_{i=1}^{N-m+1}\log C_{i}^{m}(r)$$

(5) Calculate the approximate entropy:

(7) $$\mathrm{ApEn}(m,r,N)=\Phi^{m}(r)-\Phi^{m+1}(r)$$

In our calculation, *m* was set to 2 and *r* was chosen as 20% of the standard deviation of heartbeat intervals. This selection aligns with established practices for clinical heart rate data, providing a balance between sensitivity and robustness [44].

### 3.6. Correlation Computation

After calculating the ApEn values, we determined the correlation between these entropy values and post-delivery blood gas parameters by employing Pearson’s correlation coefficient, as defined in (8):

(8) $$\hat{r}=\frac{\sum \left(x_{i}-\bar{x})(y_{i}-\bar{y}\right)}{\sqrt{\sum (x_{i}-\bar{x}{)}^{2}\sum (y_{i}-\bar{y}{)}^{2}}}$$

where $\hat{r}$ is the correlation coefficient, $x_{i}$ and $\bar{x}$ represent the calculated approximate entropy values and their averages, respectively, $y_{i}$ and $\bar{y}$ represent the corresponding blood gas parameters and their average. This analysis quantitatively assesses the relationship between the complexity of FHRV, as indicated by ApEn, and fetal health status, as reflected by blood gas measures. Understanding this relationship aids in evaluating the potential of ApEn as a predictive marker for fetal well-being.

### 3.7. Hidden Markov Model (HMM) Construction

**Figure 7:**
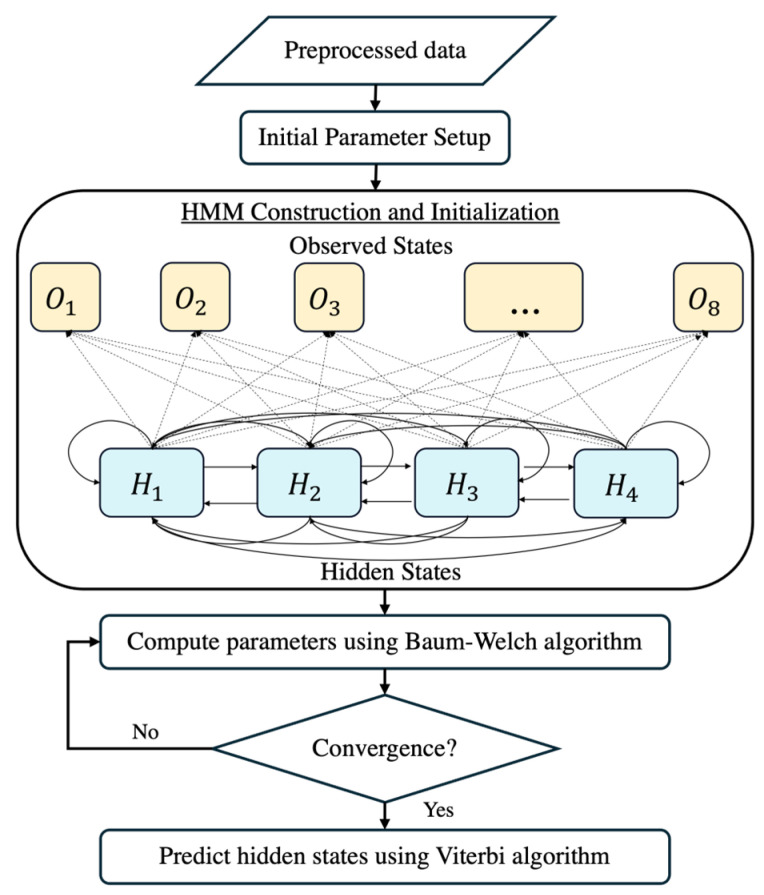
The flowchart of the HMM construction.

In this study, we employed an HMM to predict the fetal acid–base status during labor by analyzing FHRV data. The HMM is a powerful machine learning algorithm frequently applied in time-series analysis due to its ability to model systems with hidden states, influencing observable events. The overall workflow of the HMM is provided in Figure 7. Given the extensive heart rate data collected, addressing challenges associated with big data analytics is essential. To tackle these challenges, we leveraged optimized computational techniques and big data optimization methods [45].

#### 3.7.1. Hidden States

The hidden states $S=\left\{H_{1},H_{2},H_{3},H_{4}\right\}$ represent the fetal acid–base status, categorized into four discrete pH levels based on umbilical cord blood gas analysis immediately after delivery, as defined in Table 1. This categorization aligns with clinically significant pH thresholds, allowing the model to reflect meaningful physiological states.

#### 3.7.2. Observed States

The observed states $O=\left\{O_{1},O_{2},\ldots ,O_{8}\right\}$ are derived from FHRV data by analyzing the differences between consecutive bpm measurements. Initially, we calculated the difference between each pair of consecutive bpm readings to generate a time series of bpm differences. Based on these differences, we classified the data into three primary categories: Decrease (*D*), Stable (*S*), and Increase (*I*), with thresholds as follows:

- Decrease (*D*): Differences less than or equal to −2 bpm;
- Stable (*S*): Differences between −2 bpm and 2 bpm;
- Increase (*I*): Differences greater than or equal to 2 bpm.

To capture finer distinctions, we further subdivided each category into two subgroups based on the midpoint of the range of differences, resulting in six observed subgroups: *D*_1_, *D*_2_, *S*_1_, *S*_2_, *I*_1_, and *I*_2_. By generating all possible combinations of these subgroups, we created the observed states $O_{i}(i=1,2,\ldots ,8)$, as described in Table 2.

#### 3.7.3. HMM Parameters

The three probability measures are the state transition probability distribution *A*, the emission probability distribution *B*, and the initial state distribution *π*. These are the essential components of the HMM and are defined as follows:

(9) $$A=\left\{a_{ij}\right\}\;\mathrm{where}\;a_{ij}=P(H_{t+1}=H_{j}|H_{t}=H_{i})$$

(10) $$B=\left\{b_{j}(k)\right\}\;\mathrm{where}\;b_{j}(k)=P(O_{t}=O_{k}|H_{t}=H_{j})$$

(11) $$\pi =\left\{\pi_{j}\right\}\;\mathrm{where}\;\pi_{i}=P(H_{1}=H_{i})$$

(12) $$\lambda =\left(A,B,\pi \right)$$

where $a_{ij}$ is the probability of transitioning between hidden states, $b_{j}(k)$ is the probability of observing symbol $O_{k}$ at time *t* when the system is in hidden states $H_{j}$, $\pi_{i}$ is the initial probability of each hidden states, and *λ* is the complete HMM.

Regarding the initial state distribution *π*, we assume equal probabilities for each hidden state in (13), reflecting an unbiased prior state with no preference for any fetal acid–base status at the onset. This assumption is appropriate given the lack of prior information about the initial state in our context.

(13) $$\pi =\left[\begin{array}{cccc} 0.25 & 0.25 & 0.25 & 0.25 \end{array}\right]$$

#### 3.7.4. Parameter Estimation

To calculate the initial state transition probability matrix *A*, we utilized a long-duration training sequence by creating a co-occurrence matrix of transitions between hidden states, represented as (i,j) pairs, where *i* and *j* denote the previous and next hidden states, respectively. The transitions were counted across the training dataset, and probabilities were estimated by normalizing the counts for each state.

The emission probability matrix *B* was estimated using four different training and testing datasets corresponding to the hidden classes. Each dataset was divided into training and testing subsets to ensure the robust estimation of emission probabilities and to evaluate the model’s performance on unseen data. The emission probabilities $b_{j}(k)$ were calculated based on the frequency of observing each symbol $O_{k}$ when the system was in a hidden state $H_{j}$, as described in (10).

#### 3.7.5. Training and Decoding

The HMM procedure involves two critical phases: training and decoding. After initializing the state distribution π and estimating matrices *A* and *B*, we trained the model using the Baum–Welch algorithm (Algorithm 1) [45]. This algorithm iteratively refines the parameters to better fit the observed FHRV sequences, ensuring that the model accurately captures the underlying patterns associated with different fetal acid–base statuses.
**Algorithm 1:** Baum–Welch algorithm**Input**: Initial parameters: λ=(A,B,π) **Observation sequence**: $O=\left\{O_{1},O_{2},\ldots ,O_{8}\right\}$ **Output**: Updated parameters = $\hat{\lambda }=\left(\hat{A},\hat{B},\hat{\pi }\right)$ that maximize the likelihood of the probability $P\left(\lambda |O\right)$. 1. **Initialization**: $\alpha_{1}(i)=\pi_{i}b_{i}\left(O_{1}\right),\;\beta_{T}(i)=1,\;\mathrm{for}\;1\leq i\leq 4$ 2. **repeat** 3. **Expectation step (E step)** 4. **Forward–Backward recursion**: $\;\alpha_{t+1}(i)=b_{i}\left(O_{t}\right)\sum_{j=1}^{4}\alpha_{t-1}\left(j\right)a_{ji},\;1\leq i\leq 4$ $\;\beta_{t}(i)=\sum_{j=1}^{4}a_{ij}b_{j}\left(O_{t+1}\right)\beta_{t+1}\left(j\right),\;1\leq i\leq 4,T-1\leq t\leq 1$ 5. **Compute state transition probability** $\xi_{t}\left(i,j\right)$ and **state occurrence probability**
$\gamma_{t}(i)$: $\;\xi_{t}\left(i,j\right)=\frac{\alpha_{t}(i)a_{ij}b_{j}\left(O_{t+1}\right)\beta_{t+1}(j)}{\sum_{i=1}^{4}\sum_{j=1}^{4}\alpha_{t}(i)a_{ij}b_{j}\left(O_{t+1}\right)\beta_{t+1}(j)},1\leq i,j\leq 4,1\leq t\leq T-1$ $\;\gamma_{t}(i)=\frac{\alpha_{t}(i)\beta_{t}(i)}{\sum_{k=1}^{4}\alpha_{t}(k)\beta_{t}(k)},1\leq i\leq 4,1\leq t\leq T$ 6. **Maximization step** (M step) 7. **Calculate updated model parameters**:  Transition probability: ${\hat{a}}_{ij}=\frac{\sum_{t=1}^{T-1}\xi_{t}\left(i,j\right)}{\sum_{t=1}^{T-1}\gamma_{t}(i)}$  Emission probability: ${\hat{b}}_{j}(k)=\frac{\sum_{t=1}^{T}\delta \left(O_{t},O_{m}\right)\gamma_{t}(j)}{\sum_{t=1}^{T}\gamma_{t}(j)},\delta \left(O_{t},O_{m}\right)=1\;\mathrm{if}\;O_{t}=O_{m},\;\mathrm{else}\;0.$  Initial probability: ${\hat{\pi }}_{i}=\gamma_{1}(i)$ 8. **Set**
$\lambda \to \hat{\lambda }$ 9. **until the change in likelihood**
$P\left(\lambda |O\right)$
**between iteration is stable**.

Once the HMM parameters were sufficiently trained, the Viterbi algorithm (Algorithm 2) was applied to the testing dataset to decode the most probable sequence of hidden states corresponding to the observed FHRV data [45]. This decoding step enabled us to predict the fetal acid–base status in real time during labor, providing valuable insights for clinical decision-making. By identifying the sequence of hidden states that best explains the observed heart rate variability, the Viterbi algorithm allowed for timely and accurate assessments of fetal well-being. The combination of the Baum–Welch algorithm for parameter optimization and the Viterbi algorithm for state decoding ensured that our HMM-based approach is both robust and effective in predicting critical health indicators from complex, high-dimensional data.
**Algorithm 2:** Viterbi algorithm**Input**: Trained parameters $\hat{\lambda }$ with hidden states $S=\left\{H_{1},H_{2},H_{3},H_{4}\right\}$ **Observation sequence**: $O=\left\{O_{1},O_{2},\ldots ,O_{8}\right\}$ **Output**: The most probable sequence of hidden states $Q=\left\{q_{1},q_{2},\ldots ,q_{T}\right\}$. 1. **Initialization**: $\delta_{1}(i)=\pi_{i}b_{i}\left(O_{1}\right),\psi_{1}(i)=0,1\leq i\leq 4$. 2. **Recursion**: For 1≤i≤4,2≤t≤T, $\;\delta_{t}(i)=\underset{1\leq j\leq 4}{\max}\left[\delta_{t-1}(j)a_{ji}\right]b_{i}\left(O_{t}\right),\psi_{t}(i)=\arg\underset{1\leq j\leq 4}{\max}\left[\delta_{t-1}(j)a_{ji}\right]$ 3. **Termination**: $P^{*}=\underset{1\leq i\leq 4}{\max}\delta_{T}(i),q_{T}^{*}=\arg\underset{1\leq i\leq 4}{\max}\delta_{T}(i)$ 4. **Path backtracking**: $q_{t}^{*}=\psi_{t+1}\left(q_{t+1}^{*}\right),1\leq t\leq T-1$ 5. **return the most probable sequence** Q

## 4. Experimental Results and Discussion

In this section, we present the dataset, results, and discussion of our experimental analysis. The results highlight the effectiveness of our methods in distinguishing between parasympathetic and sympathetic activity, followed by an in-depth discussion of the findings and their implications for fetal monitoring. Additionally, we demonstrate how the HMM effectively predicts the fetal acid–base status based on heart rate variability patterns.

### 4.1. Dataset

Our study utilized a dataset comprising FHR data obtained through fetal electrocardiogram (ECG) recordings. The initial dataset consisted of 585 recordings of the FHR measured in beats per minute (bpm). To ensure the quality and consistency of the data, we selected recordings with a duration of more than one hour, resulting in a final dataset of 469 FHR recordings for analysis. This selection criterion was applied to capture sufficient temporal information for reliable modeling and to enhance the robustness of our entropy and HMM analysis. Ethical approval for the use of fetal ECG data was obtained from the relevant institutional review board, and all data were anonymized to protect patient confidentiality.

### 4.2. Entropy Analysis

**Figure 8:**
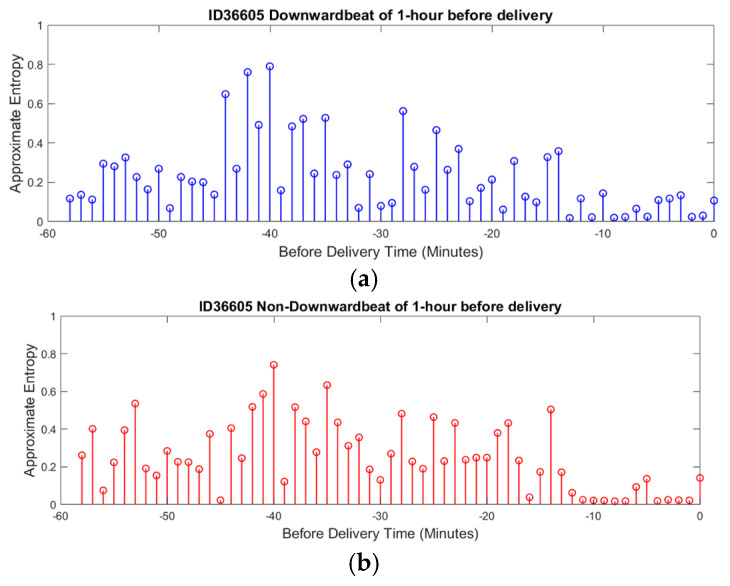
ApEn of FHRV during delivery time. (**a**) ApEn during downward beat (Parasympathetic). (**b**) ApEn during non-downward beat (Sympathetic).

The average ApEn for downward beats across all recordings was 0.85 ± 0.10, while for non-downward beats, it was 0.78 ± 0.12. The statistical analysis using a paired *t*-test showed that the difference between the two beat types was significant (*p*-value < 0.05), suggesting greater complexity in parasympathetic activity. In the context of downward beats, as shown in Figure 8a, ApEn fluctuates over time, with alternating periods of higher complexity and lower complexity. Higher ApEn values indicate increased irregularity and complexity in FHRV, which may reflect a healthy and responsive autonomic nervous system. Conversely, a gradual reduction in ApEn approaching delivery could signal fetal adaptation to stress or potential distress, warranting closer monitoring.

A noticeable trend is the gradual reduction in complexity as delivery approaches, suggesting a possible adaptation mechanism in response to increasing physiological stress. This sample patient data illustrate how ApEn is utilized in this research to reveal patterns of physiological adaptation and stress regulation, demonstrating its potential to provide valuable insights into fetal well-being.

For non-downward beats, ApEn shows similar patterns of fluctuation but with subtle differences in the distribution and timing of peaks and valleys, as shown in Figure 8b. This suggests that different beat categories may correspond to unique physiological states or responses. The dynamic nature of ApEn for non-downward beats, with periods of higher complexity potentially indicating stable or compensatory responses, further highlights the usefulness of this measure in assessing fetal well-being.

In clinical practice, the use of ApEn offers a comprehensive view of fetal well-being by providing detailed information on autonomic nervous system activity. Through scalp electrode measurements integrated into a virtual replica of a digital twin model, this analysis can simulate the functioning of each branch of the nervous system in real time. This capability ensures that approximate entropy not only tracks dynamic changes in FHRV but also provides a holistic picture of fetal physiology.

### 4.3. Correlation Analysis Outcome

The relationship between FHRV during delivery and umbilical cord blood gas parameters after delivery was analyzed to assess the clinical relevance of our approach. Table 3 presents the correlation coefficients between approximate entropy (ApEn) and umbilical cord blood parameters, including pH, base excess, and lactate levels. These parameters are critical indicators of the fetal acid–base status and overall well-being. Among these, ApEn showed the strongest positive correlation with pH values, particularly for parasympathetic activity, while correlations with other parameters such as base excess and lactate were weaker but still noteworthy. Moreover, this correlation suggests that higher ApEn values are associated with a better acid–base status in the fetus. This relationship underscores the potential of ApEn as a non-invasive indicator for anticipating prenatal outcomes, allowing for earlier interventions during labor.

**Figure 9:**
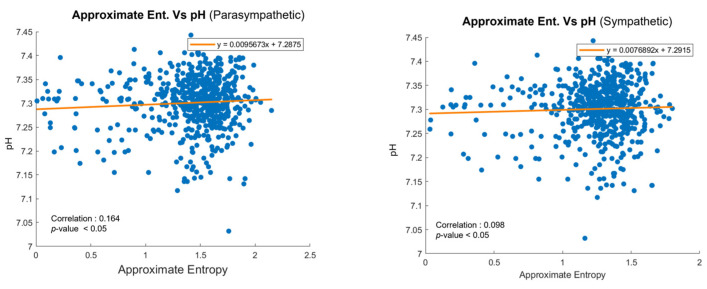
Scatter plots of ApEn and pH values for (**a**) parasympathetic and (**b**) sympathetic activity.

Figure 9 illustrates the correlation between ApEn and pH values for parasympathetic and sympathetic nervous system activity, respectively [4]. In the parasympathetic branch shown in Figure 9a, a modest positive correlation is observed, suggesting that higher complexity in FHR patterns may be linked to a better acid–base status. Similarly, for sympathetic activity, shown in Figure 9b, a smaller yet significant positive correlation was found. Given that FHR data are collected during delivery and pH values are measured after delivery, the observed correlations highlight the utility of ApEn in bridging these time-separated datasets. Each dot in the graph represents an individual patient, emphasizing the personalized nature of the dataset. By identifying the associations between real-time FHR variability and post-delivery outcomes, this analysis supports the integration of ApEn into predictive models, such as HMMs, for enhanced fetal monitoring.

### 4.4. Hidden Markov Model (HMM) Evaluation

**Figure 10:**
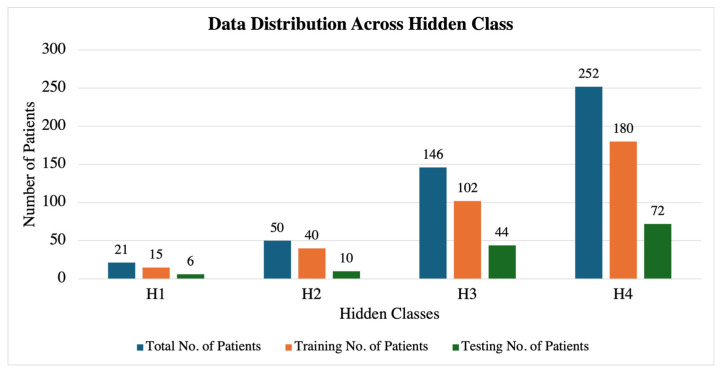
Dataset distribution across each hidden class.

Building upon the insights gained from the entropy analysis and correlation studies, we employed an HMM to predict the fetal acid–base status based on heart rate variability patterns observed in the hour preceding delivery. For the HMM analysis, we categorized the fetal acid–base status into four hidden states based on umbilical cord blood pH values from the total of 469 fetal datasets, as shown in Table 4 and Figure 10. The pH thresholds were chosen in consultation with medical professionals to reflect clinically significant ranges, with each group differing by 0.05 units. This partitioning aligns with established clinical guidelines and provides meaningful distinctions between different levels of the fetal acid–base status. The 0.05 pH increments are significant in prenatal care, as small changes in pH can indicate substantial physiological differences in the fetus, influencing clinical decision-making and interventions. The increasing accuracy with longer recording durations suggests that the HMM effectively captures the temporal dependencies and patterns associated with the fetal acid–base status. The overlapping prediction approach provides valuable insights into how reliable predictions can be made early in the labor process. The early detection of potential fetal distress is crucial for timely clinical interventions.

To evaluate the performance of our HMM in predicting the fetal acid–base status, we calculated the accuracy of the model’s final predictions by comparing them with the actual pH values obtained from the umbilical cord blood gas analysis. The accuracy was assessed using confusion matrices for both the training and testing datasets, which provide detailed insights into the model’s classification capabilities across the four hidden states.

In the confusion matrix of the training patients’ dataset, which is described in Table 5, our HMM demonstrated strong classification performance for the normal fetal acid–base status, correctly identifying most instances in state H_3_ and H_4_. This indicates the model’s capability in recognizing normal or near-normal pH levels with high reliability. However, the classification accuracy for states associated with fetal acidosis was lower, with several H_1_ and H_2_ instances misclassified as higher pH states (H_3_ or H_4_). Specifically, misclassifications occurred where H_1_ was often predicted as H_3_, and H_2_ was frequently classified as H_3_ or H_4_. This suggests that the model struggles to differentiate between mild and severe acidosis. These misclassification patterns highlight the need for further refinement in distinguishing lower pH levels, possibly by incorporating additional physiological parameters or adjusting the class balance in training.

In the confusion matrix of testing patients’ accuracy shown in Table 6, the model maintained strong classification performance for the normal fetal acid–base status, correctly identifying most instances in state H_3_ and H_4_. This demonstrates its ability to generalize well to unseen data for these states. However, misclassifications were observed, particularly with H_1_ being classified as H_2_, H_3_, or H_4_, and H_2_ frequently predicted as H_3_ or H_4_. These misclassification patterns suggest that overlapping FHRV features between these states, as well as limited training data for H_1_ and H_2_, may contribute to the model’s difficulty in distinguishing lower pH levels. Addressing these challenges through additional training data or feature enhancement may improve classification performance for fetal acidosis cases.

Table 7 and Table 8 present the classification performance of the HMM model for the training and testing datasets, respectively, evaluating sensitivity, specificity, precision, F1-score, and accuracy for each hidden class. The model performs well in classifying the normal fetal acid–base status in both datasets, with relatively high sensitivity and precision. However, classification performance for fetal acidosis cases (H_1_ and H_2_) remains low, with sensitivity values of 0.33 and 0.43 in training, as shown in Table 7, and 0.33 and 0.10 in testing, as shown in Table 8, indicating a persistent difficulty in distinguishing between low pH states. The drop in H_2_ sensitivity from 0.43 in training to 0.10 in testing suggests that the model struggles to generalize well for this class, potentially due to feature overlap with normal states or insufficient training samples for acidosis cases. While specificity remains high for all classes, the misclassification of H_1_ and H_2_ into higher pH states contributes to reduced sensitivity, limiting the model’s ability to detect fetal distress accurately. The overall accuracy decreases from 78% in training to 71% in testing, highlighting a slight performance degradation when applied to unseen data. These findings highlight the need for further enhancements, including advanced feature engineering, data balancing techniques, and refined classification methodologies, to improve the model’s ability to accurately distinguish borderline acid–base status classifications.

These experimental results demonstrate the effectiveness of integrating approximate entropy analysis and HMM within a digital twin framework for predicting fetal acid–base status. The findings suggest that this approach has the potential to enhance real-time fetal monitoring and support timely clinical interventions.

### 4.5. Discussion

Integrating the HMM-based predictive model within the digital twin framework enhances real-time fetal monitoring by continuously updating fetal cardiovascular dynamics and predicting acid–base status changes with improved accuracy. Approximate entropy measures further refine these predictions by capturing complex variations in FHRV, offering a more nuanced assessment of fetal well-being. The digital twin serves as a dynamic virtual representation of the fetal cardiovascular system, allowing for the simulation of various physiological conditions and responses. By incorporating approximate entropy measures into the digital twin, we capture the complexity of FHRV and provide a more comprehensive assessment of fetal well-being. This integration enables clinicians to visualize and predict fetal responses to different stressors or interventions, offering a level of personalized care not achievable with traditional monitoring methods.

A notable limitation in our initial study was the uneven distribution of datasets among the hidden states, with the H_1_ and H_2_ groups comprising approximately 15% of the total data. This imbalance resulted in fewer examples for the model to learn the patterns associated with fetal acidosis, potentially leading to overfitting on the limited samples and insufficient generalization to new data. Similar issues have been noted in other studies, where a class imbalance affects model performance [46].

To address this limitation, we conducted additional experiments using a balanced dataset comprising twenty-one randomly chosen samples for each hidden state (H_1_, H_2_, H_3_, H_4_), with fifteen samples used for training and six for testing in each category. This balanced approach aimed to provide the HMM with an equal representation of all classes, thereby enhancing its ability to learn and generalize across different fetal pH values.

Table 9 and Table 10 present the confusion matrices for the model’s classification performance in training and testing, evaluating its ability to distinguish between the four fetal acid–base status categories using the same dataset distribution. In Table 9, the model demonstrates strong classification performance for H_3_ and H_4_, with most instances correctly predicted within their respective categories. However, misclassifications occur in H_1_ and H_2_, where H_1_ is sometimes predicted as H_2_ or H_3_, and H_2_ is occasionally classified as H_3_. This pattern suggests that the model struggles to differentiate between fetal acidosis states and higher pH conditions. Similarly, Table 10 follows the same dataset structure but shows a different prediction distribution. The model still performs well in identifying H_3_ cases, with most instances correctly classified. However, H_1_ and H_4_ exhibit notable misclassification errors, with H_1_ often predicted as H_2_ or H_3_, and H_4_ showing some confusion with H_1_ and H_2_. These trends indicate that while the model maintains stability across datasets, it faces challenges in accurately identifying fetal acidosis cases, which are often misclassified as higher pH states.

The balanced dataset considerably improved the model’s ability to detect fetal acidosis cases, addressing the misclassification issues observed in the imbalanced dataset. Previously, the model tended to classify lower pH states as normal conditions due to class dominance. By equalizing the dataset, the model learned to differentiate acidotic cases more effectively, reducing false predictions of fetal distress as normal states. In the confusion matrices of the imbalanced dataset in Table 5 and Table 6, the model tends to misclassify H_1_ and H_2_ as higher pH states, likely due to class dominance influencing decision boundaries. However, in Table 9 and Table 10, misclassification is more evenly distributed, reducing the tendency to predict acidosis cases as normal. This suggests that class imbalance contributes to model bias, leading to the overrepresentation of dominant classes in predictions, whereas balancing the dataset helps stabilize classification boundaries. Although improvements are evident, some shifts in misclassification patterns, particularly in H_4_, indicate that further refinements, such as feature selection and additional physiological parameters, may enhance the model’s ability to generalize across varying dataset distributions.

Table 11 and Table 12 present the classification performance of the HMM model using a balanced dataset for training and testing, respectively. In Table 11, the model demonstrates high sensitivity across all classes, with H_1_ and H_2_ achieving 0.80 and 0.93, respectively, indicating a substantial improvement in detecting fetal acidosis cases. Specificity remains consistently high across all classes, ranging from 0.88 to 0.97, ensuring that false positives are minimized. Notably, the model maintains strong classification performance for the normal fetal acid–base status (H_3_ and H_4_), with an F1-score exceeding 0.82 in all categories. The overall accuracy of 85% suggests that the model effectively learns from the balanced dataset, leading to more stable predictions across all classes.

In the testing dataset in Table 12, the model continues to perform well, with an overall accuracy of 79%. Sensitivity for H_1_ and H_2_ remains relatively high at 0.66 and 0.83, respectively, though slightly lower than in training, indicating a moderate generalization gap. The model exhibits strong classification performance for H_3_ (sensitivity = 1.00) and H_4_ (specificity = 1.00), ensuring that normal fetal conditions are accurately distinguished from acidosis cases. While precision and F1-scores remain high, slight fluctuations suggest that further refinement in model training or feature selection could enhance generalization performance.

When compared to the imbalanced dataset performance in Table 7 and Table 8, the balanced dataset significantly improves the classification of H_1_ and H_2_, while maintaining strong performance for H_3_ and H_4_. Sensitivity for H_1_ and H_2_ improved from 0.33 and 0.43 in Table 7 to 0.80 and 0.93 in Table 11, demonstrating a substantial enhancement in detecting fetal acidosis cases. Similarly, in Table 8, the sensitivity of H_1_ and H_2_ increased from 0.33 and 0.10 to 0.66 and 0.83 in Table 12, confirming that class balancing significantly mitigated bias toward normal conditions and improved model robustness in identifying acidotic states. This indicates that the imbalanced class distribution in the original dataset led to model bias toward the majority classes, whereas balancing mitigated this effect, allowing for the better detection of fetal acidosis cases. Similarly, in Table 8, H_1_ and H_2_ sensitivities were 0.33 and 0.10, respectively, which improved to 0.66 and 0.83 in Table 12, confirming a substantial enhancement in detecting lower pH states. Moreover, the overall accuracy improved from 78% to 85% in training and from 71% to 79% in testing, reflecting better generalization when using a balanced dataset. However, despite these improvements, minor variations in specificity and F1-scores suggest that additional fine-tuning, such as alternative balancing methods or feature engineering, may further optimize classification performance across different fetal acid–base states.

While achieving higher accuracy remains challenging due to the complexity of FHRV patterns and the subtle distinctions between acid–base statuses, future work will focus on enhancing the model’s predictive capability for fetal acidosis. Expanding the dataset for underrepresented classes, along with implementing techniques such as data augmentation, synthetic minority over-sampling (SMOTE), and class weighting, will help mitigate class imbalance and improve classification robustness [47].

While the achieved accuracy is not as high as some structured medical studies, fetal heart rate variability data are highly complex, influenced by multiple physiological factors, and subject to significant noise. Future improvements may include incorporating additional clinical parameters or advanced feature engineering techniques to enhance predictive performance.

By continuously integrating real-time FHRV data, the digital twin model provides actionable insights into fetal well-being, allowing clinicians to anticipate distress patterns and take preventive measures before complications arise. The model’s ability to dynamically simulate physiological responses enhances clinical decision-making, supporting early intervention and personalized maternal–fetal care. In a clinical setting, such insights could prompt obstetricians to take preventive actions, such as closer monitoring, maternal repositioning, or early intervention, to mitigate potential adverse outcomes. By providing a real-time, data-driven decision support system, the digital twin model has the potential to enhance the early detection of fetal distress and improve maternal–fetal care in the future. Furthermore, the use of a digital twin allows for personalized fetal monitoring by simulating individual physiological responses in real time. This personalization enhances the accuracy of predictions and supports tailored clinical care. By leveraging existing ECG monitoring technology and advanced analytics, our system can be seamlessly integrated into current clinical workflows without requiring significant additional resources or training. Ultimately, the consumer benefits of our system lie in its potential to improve maternal and fetal health outcomes through advanced, data-driven fetal monitoring.

## 5. Conclusions

In this study, we developed an advanced predictive analytics approach by integrating approximate entropy analysis with an HMM within a digital twin framework to enhance real-time fetal monitoring. Our model effectively captures temporal dependencies and provides early predictions of the fetal acid–base status based on FHRV patterns. The experimental results demonstrated high accuracy in predicting normal pH levels, indicating the model’s strong capability in identifying normal or near-normal fetal conditions.

When compared to a previous study, in which a support vector machine (SVM) and genetic algorithm (GA)-based approach was used [27], our model provides a more nuanced, multi-state classification rather than a binary classification. Unlike traditional machine learning models that rely on static feature selection, our approach dynamically tracks state transitions over time, providing real-time adaptability to evolving fetal conditions. This ability enhances continuous fetal monitoring and supports early clinical intervention strategies by offering a more detailed, multi-state classification of the fetal acid–base status. The digital twin model provides a comprehensive physiological assessment, which is crucial for early intervention and continuous monitoring.

The integration of this predictive model into clinical practice has the potential to significantly improve maternal and fetal health outcomes through proactive and personalized care. By providing immediate, non-invasive insights into the fetal condition during labor, our system enables timely and informed decision-making, facilitating early interventions that can reduce the risk of complications and improve prenatal outcomes. Future research will focus on expanding the dataset for underrepresented classes and implementing advanced modeling techniques to improve the detection of fetal distress. The successful integration of this predictive model into clinical practice holds the potential to significantly enhance maternal and fetal health outcomes through timely and informed interventions.

## Figures and Tables

**Table 1:** Classification of hidden states.

Hidden Class (H)	Condition
H_1_	pH<7.2
H_2_	7.2≤pH <7.25
H_3_	7.25≤pH <7.23
H_4_	pH≥7.3

**Table 2:** Classification of observed states.

Observed Class (*O*)	Conditional Probability
$O_{1}$	$P(D_{1}|D)\times P(S_{1}|S)\times P(I_{1}|I)$
$O_{2}$	$P(D_{1}|D)\times P(S_{1}|S)\times P(I_{2}|I)$
$O_{3}$	$P(D_{1}|D)\times P(S_{2}|S)\times P(I_{1}|I)$
$O_{4}$	$P(D_{1}|D)\times P(S_{2}|S)\times P(I_{2}|I)$
$O_{5}$	$P(D_{2}|D)\times P(S_{1}|S)\times P(I_{1}|I)$
$O_{6}$	$P(D_{2}|D)\times P(S_{1}|S)\times P(I_{2}|I)$
$O_{7}$	$P(D_{2}|D)\times P(S_{2}|S)\times P(I_{1}|I)$
$O_{8}$	$P(D_{2}|D)\times P(S_{2}|S)\times P(I_{2}|I)$

**Table 3:** Correlation between ApEn of FHRV and blood gas parameters.

Method	pH	PCO_2_	PO_2_	HCO_3_	BE
Downward (Parasympathetic)	0.164	0.067	0.052	0.104	0.057
Non-downward (Sympathetic)	0.098	0.054	0.011	0.064	0.032

**Table 4:** Distribution of datasets for HMM analysis.

Hidden Class	Category	Total No. of Patient	No. of Training	No. of Testing
H_1_	pH<7.2	21	15	6
H_2_	7.2≤pH <7.25	50	40	10
H_3_	7.25≤pH <7.25	146	102	44
H_4_	pH≥7.3	252	180	72

**Table 5:** Confusion matrix with accuracy on training patients’ data.

Actual	Predicted			
	H_1_	H_2_	H_3_	H_4_
H_1_	5	2	6	2
H_2_	4	17	8	11
H_3_	0	2	85	15
H_4_	0	3	21	156

**Table 6:** Confusion matrix with accuracy on testing patients’ data.

Actual	Predicted			
	H_1_	H_2_	H_3_	H_4_
H_1_	2	1	1	2
H_2_	1	1	5	3
H_3_	0	2	32	10
H_4_	0	1	12	59

**Table 7:** Performance metrics of hidden Markov model (HMM) for training patients’ data.

Class	Sensitivity	Specificity	Precision	F1-Score	Accuracy
H_1_	0.33	0.98	0.56	0.42	0.95
H_2_	0.43	0.97	0.71	0.53	0.91
H_3_	0.83	0.85	0.71	0.77	0.85
H_4_	0.87	0.82	0.85	0.86	0.85
Overall accuracy	78%				

**Table 8:** Performance metrics of hidden Markov model (HMM) for testing patients’ data.

Class	Sensitivity	Specificity	Precision	F1-Score	Accuracy
H_1_	0.33	0.99	0.66	0.44	0.96
H_2_	0.10	0.96	0.20	0.13	0.90
H_3_	0.73	0.79	0.64	0.68	0.77
H_4_	0.81	0.75	0.79	0.80	0.78
Overall accuracy	71%				

**Table 9:** Confusion matrix with accuracy on training balanced dataset.

Actual	Predicted			
	H_1_	H_2_	H_3_	H_4_
H_1_	12	2	1	0
H_2_	1	14	0	0
H_3_	0	2	12	1
H_4_	0	1	1	13

**Table 10:** Confusion matrix with accuracy on testing balanced dataset.

Actual	Predicted			
	H_1_	H_2_	H_3_	H_4_
H_1_	4	1	1	0
H_2_	1	5	0	0
H_3_	0	0	6	0
H_4_	1	1	0	4

**Table 11:** Performance metrics of hidden Markov model (HMM) for training balanced dataset.

Class	Sensitivity	Specificity	Precision	F1-Score	Accuracy
H_1_	0.80	0.97	0.92	0.86	0.93
H_2_	0.93	0.88	0.73	0.82	0.90
H_3_	0.80	0.95	0.85	0.82	0.91
H_4_	0.86	0.97	0.92	0.89	0.95
Overall accuracy	85%				

**Table 12:** Performance metrics of hidden Markov model (HMM) for testing balanced dataset.

Class	Sensitivity	Specificity	Precision	F1-Score	Accuracy
H_1_	0.66	0.94	0.80	0.72	0.87
H_2_	0.83	0.88	0.71	0.77	0.87
H_3_	1.00	0.94	0.85	0.92	0.96
H_4_	0.67	1.00	1.00	0.80	0.92
Overall accuracy	79%				

## Data Availability

The data presented in this study are available on request from the corresponding author.
